# On Complexities of Impact Simulation of Fiber Reinforced Polymer Composites: A Simplified Modeling Framework

**DOI:** 10.1155/2014/382525

**Published:** 2014-11-10

**Authors:** M. Alemi-Ardakani, A. S. Milani, S. Yannacopoulos

**Affiliations:** School of Engineering, University of British Columbia, Kelowna, BC, Canada V1V 1V7

## Abstract

Impact modeling of fiber reinforced polymer composites is a complex and challenging task, in particular for practitioners with less experience in advanced coding and user-defined subroutines. Different numerical algorithms have been developed over the past decades for impact modeling of composites, yet a considerable gap often exists between predicted and experimental observations. In this paper, after a review of reported sources of complexities in impact modeling of fiber reinforced polymer composites, two simplified approaches are presented for fast simulation of out-of-plane impact response of these materials considering four main effects: (a) strain rate dependency of the mechanical properties, (b) difference between tensile and flexural bending responses, (c) delamination, and (d) the geometry of fixture (clamping conditions). In the first approach, it is shown that by applying correction factors to the quasistatic material properties, which are often readily available from material datasheets, the role of these four sources in modeling impact response of a given composite may be accounted for. As a result a rough estimation of the dynamic force response of the composite can be attained. To show the application of the approach, a twill woven polypropylene/glass reinforced thermoplastic composite laminate has been tested under 200 J impact energy and was modeled in Abaqus/Explicit via the built-in Hashin damage criteria. X-ray microtomography was used to investigate the presence of delamination inside the impacted sample. Finally, as a second and much simpler modeling approach it is shown that applying only a single correction factor over all material properties at once can still yield a reasonable prediction. Both advantages and limitations of the simplified modeling framework are addressed in the performed case study.

## 1. Introduction

Finite element analysis (FEA) has been employed in a large portion of past investigations on modeling and predicting the response of fiber reinforced composite materials. To give a few examples, a three-dimensional computational micromechanical model was developed for woven fabric composites by Ivanov and Tabiei [1]. The impact response of unidirectional composite laminates was modeled by Aminjikarai and Tabiei [2] using a strain-rate dependent micromechanical model with a progressive damage behavior. Petrossian and Wisnom [3] developed interface elements to create a resin-rich area between plies to predict the onset and growth of delamination in composite laminates. Atas et al. [4] used cohesive zone elements with a bilinear traction-separation law to predict the delamination initiation and growth in pin-loaded composite laminates. A finite element model was developed by Komeili and Milani [5] to consider the effect of meso-level uncertainties on the mechanical response of axially loaded woven composites. Next to modeling efforts, several impact and post-impact tests have also been conducted on composites (e.g., [6–8]) to understand damage mechanisms experimentally and validate the associated finite element codes. Despite these efforts, a fully representative numerical model has not been developed to date to predict composites response under all different impact conditions, or it would be computationally very expensive.

Fiber reinforced plastic (FRP) composites are in particular known to be difficult materials to be modelled numerically because of their nonlinear and occasionally nonrepeatable mechanical responses under high velocity events. Sources of difference between their numerical modeling and experimental results are attributed to, on one hand, various uncertain parameters in the material (such as fiber misalignment/waviness, voids, and nonuniform volume fraction distribution), and on the other hand, modeling errors (such as assumptions made in fiber-matrix bonding behavior, rate—and deformation mode—dependency of the material parameters, etc.). Factors contributing to the latter category (modeling complexities) will be reviewed in detail in Section 4. Multiscale nature of composites and additional uncertainty during their manufacturing (such as curing time, evenness of applied pressure and temperature throughout the part, etc.) can further add to the complexity in computational simulation of these materials.

The present work is aimed at demonstrating simplified modeling frameworks to assist practitioners in using built-in options of FEA packages (e.g., the built-in Hashin model in Abaqus) for fast simulation of high-speed impact response of composites (i.e., without a need for user-defined coding). Two approaches are suggested: (a) using different correction factors on different quasistatic properties of the material considering the effects from high strain rates, the bending mode, delamination, and clamping conditions and (b) using only a single correction factor for all the material properties simultaneously. As a sample case study, the impact behavior of a glass fiber/polypropylene thermoplastic composite has been investigated against both approaches.

## 2. Case Study Experiments

**Figure 1:**
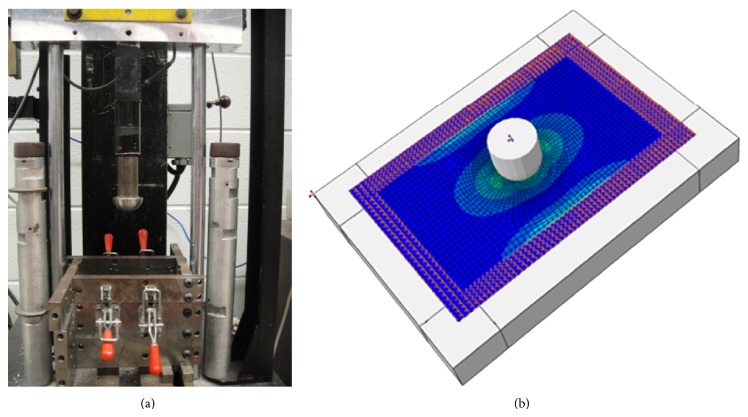
(a) Impact test set-up and (b) FE model of the drop weight test using standard composite shell for the laminate.

Composite coupons comprised of six layers of commingled glass fiber and polypropylene balanced twill weave (commercially known as Twintex) were fabricated and tested under impact according to ASTM D7136 [11]. The size of samples was 6 × 100 × 150 mm. One centimeter width of all four sides of samples was fully clamped by the test fixture shown in Figure 1(a). Impact tests were done at 200 J using the drop-weight impact test tower equipped with a one-inch diameter stainless steel hemispherical impactor. Piezoelectric sensors mounted on the impactor were used to record the reaction force exerted to the material during the collision period. The quasistatic material properties of the samples were taken from the Twintex material datasheet shown in Table 1.

## 3. Conventional Shell Finite Element Model and Limitations

The above mentioned glass fiber/PP laminates subjected to 200 J drop weight impact were simulated in Abaqus/Explicit with deformable composite shell elements as shown in Figure 1(b). The model consists of a 3D analytical rigid projectile, a deformable laminate, and an analytical rigid fixture (support). Similar to the actual test condition, the projectile was fully constrained, except in the vertical direction along which the velocity was defined at an initial value of 5.69 m/s (corresponding to a 200 J energy given the initial height of the impactor and its mass). The assigned mesh type for the multilayer composite was S4R which is a 4-node doubly curved shell element with reduced integration. The hourglass control, finite membrane strains, second order accuracy, and element deletion options were activated. The built-in Hashin progressive damage criterion was chosen to automatically decrease the mechanical properties of elements as a function of damage intensity during the impact event.

**Figure 2:**
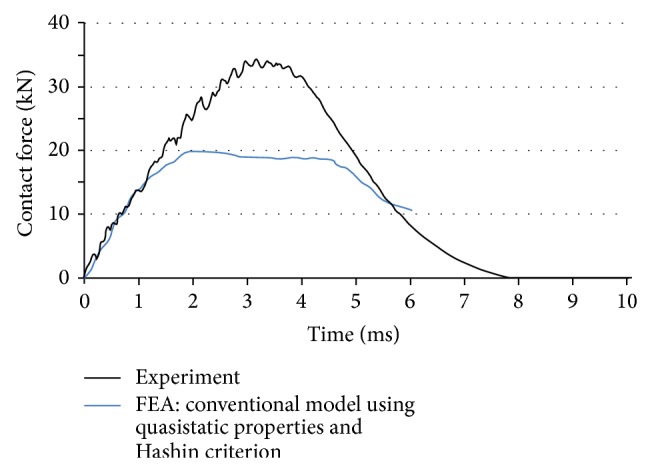
Comparison between experimental results and a conventional FEA using quasistatic properties and Hashin progressive damage criterion.

Figure 2 compares the obtained numerical and experimental results. As addressed in Section 1, different factors can play a role in causing deviation between these two responses. In particular, the effect of (a) strain rate, (b) bending mode during impact, (c) delamination, and (d) clamping system is discussed in detail in the following sections.

### 3.1. Effect of Strain Rate

The impact event modeled in the previous section relies on a dynamic deformation mode encompassing high strain rates. It is known that there is a significant difference between quasistatic and dynamic material properties of composites. Barré et al. [13] conducted a comprehensive review on relationships between applied strain rate and effective mechanical properties of a wide range of fiber-reinforced thermoset matrix composites. Their review showed that the change in the composite effective properties is dependent on the type of fibers (glass/graphite/Kevlar), the resin type (epoxy/polyester), and the induced strain rate (ranging from a quasistatic slow test to 500 s^−1^). With the exception of few cases, the general trend of increase in mechanical strength by increasing the strain rate was observed [7]. Barré et al. [13] also performed some experimental studies to investigate the effect of strain rate (10^−1^ to 10 s^−1^) on glass fiber-reinforced phenolic and polyester matrix composites. They found that the effective mechanical properties of the composites are additionally dependent on the architecture of fiber reinforcement as well as the test set-up. Foroutan et al. [14] recently studied the effect of strain rate on mechanical properties of carbon fiber-reinforced epoxy and Bismalemide (BMI) matrix composites with three different weave patterns. In general, they found that maximum tensile and shear strengths increase with the strain rate, regardless of the type of fiber architecture. In particular, they reported up to 40% and 74% increase in the tensile and shear strength properties, respectively, under dynamic events.

Although the majority of the current literature on impact response of FRPs has been devoted to thermosetting composites, some studies have been focused on thermoplastic composites. Todo et al. [15] investigated the high strain rate response of different types of fiber reinforced polyamides and found that, in general, polyamide matrix composites show an increased tensile strength and failure strain when subjected to high strain rates. Vashchenko et al. [16] reported a linear increase of tensile strength with respect to the log of strain rate in the range of 10^−3^–10^5^ s^−1^ for polyamide matrix composites. Kawata et al. [17] researched on short graphite fiber reinforced nylons and also reported an increase in the tensile strength with increasing the strain rate. Bai et al. [18] tested the high strain rate properties of high density polyethylene (HDPE) composites. Their results showed a higher Young's modulus as well as the tensile strength under high strain rate tensile loads. Papadakis et al. [19, 20] focused on continuous glass fiber/polypropylene composites (Plytron) and reported an increase in the elastic modulus and the shear strength values and a decrease in the failure strain and the shear modulus of the material as the strain rate was increased. Surprisingly, as opposed to the previous findings on other composites, the tensile strength for this material was reported to be unaffected by strain rate. McKown and Cantwell [21] studied the behavior of self-reinforced polypropylene (PP) composites under high strain rate tensions at the range of 10^−4^–10 s^−1^. They noted that initial elastic stiffness, yield strength, and tensile strength were increased with increasing the strain rate. They also reported deterioration of the failure strain by increasing the strain rate.

**Figure 3:**
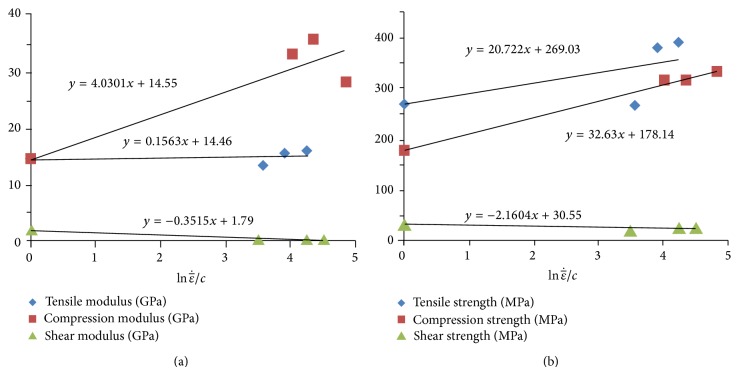
Relationship between strain rate and (a) stiffness and (b) strength for the balanced twill weave Twintex laminate (adapted from [9]).

A few earlier studies have been devoted to impact response of commingled woven glass/polypropylene thermoplastic composites. Bonnet [22] investigated the response of unbalanced woven (4 : 1) Twintex laminates subjected to a wide range of strain rates (10^−1^ to 100 s^−1^). Surprisingly, it was observed that, with increasing the strain rate, the average tensile and shear moduli decreased for this material. However, the tensile and shear strengths increased with increasing the strain rate. Finally, Brown et al. [9] studied the effect of strain rate ranging from quasistatic to 100 s^−1^ on the mechanical properties of balanced twill woven PP/glass composites (Twintex), which is also the type of material used in the present study. According to [9], the tensile and compression moduli and strengths increase with increasing the strain rate, while the shear modulus and strength show a decrease at higher strain rates. Brown et al. [9] used the logarithmic function developed by Yen and Gillespie et al. [23, 24] for their curve fitting as follows:

(1) $$\begin{array}{c} E_{RT}=E_{0}\left(1+A\ln\frac{\dot{\overset{-}{\varepsilon }}}{C}\right),\\ S_{RT}=S_{0}\left(1+B\ln\frac{\dot{\overset{-}{\varepsilon }}}{C}\right), \end{array}$$

where *E*_0_ and *E*_*RT*_ are the quasistatic and adjusted (high strain rate) moduli, *S*_0_ and *S*_*RT*_ are the quasistatic and adjusted strengths, respectively, and *A*, *B*, and *C* are constants obtained from experiments. The linear regression model between mechanical properties and the log of strain rate reported by Brown et al. [9] is summarized in Figure 3 and used in the present work during subsequent finite element models in Section 4.

### 3.2. Effect of Bending

Mechanical properties provided in material datasheets are often obtained via conventional uniaxial tension and compression tests at quasistatic rates. This is despite the fact that deformation during out-of-plane impacts is more similar to flexural bending modes than the axial tension or compression. Hallett [25] performed a statistical study on this topic and found that fiber reinforced composites show a notably higher tensile strength in the bending mode compared to standard axial tension. Further to [20], Santiuste et al. [26] were able to find a good match between their experimental and numerical results of composite laminated beams by increasing the quasistatic tensile strength by 40% in order to account for the effect of bending mode.

### 3.3. Effect of Delamination

The only failure criterion built in Abaqus explicit 6.12 for progressive damage modeling of fiber reinforced composites was the Hashin criterion. This criterion considers fiber and matrix failure in tensile and compression with the following formulations [27, 28]:

 fiber tension (${\hat{\sigma }}_{11}\geq 0$):
  (2) $$\begin{array}{c} F_{f}^{t}={\left(\frac{{\hat{\sigma }}_{11}}{X^{T}}\right)}^{2}+{\alpha \left(\frac{{\hat{\tau }}_{12}}{S^{L}}\right)}^{2}, \end{array}$$
 matrix tension (${\hat{\sigma }}_{22}\geq 0$) which here equivalently corresponds to the second fiber family tension:
  (3) $$\begin{array}{c} F_{m}^{t}={\left(\frac{{\hat{\sigma }}_{22}}{Y^{T}}\right)}^{2}+{\left(\frac{{\hat{\tau }}_{12}}{S^{L}}\right)}^{2}, \end{array}$$
 fiber compression (${\hat{\sigma }}_{11}<0$):
  (4) $$\begin{array}{c} F_{f}^{c}={\left(\frac{{\hat{\sigma }}_{11}}{X^{C}}\right)}^{2}, \end{array}$$
 matrix compression (${\hat{\sigma }}_{22}<0$):
  (5) $$\begin{array}{c} F_{m}^{c}={\left(\frac{{\hat{\sigma }}_{22}}{{2S}^{T}}\right)}^{2}+\left[{\left(\frac{Y^{C}}{{2S}^{T}}\right)}^{2}-1\right]\frac{{\hat{\sigma }}_{22}}{Y^{C}}+{\left(\frac{{\hat{\tau }}_{12}}{S^{L}}\right)}^{2}, \end{array}$$

where *X* ^*T*^, *X* ^*C*^, *Y* ^*T*^, *Y* ^*C*^, *S* ^*L*^, and *S* ^*T*^ are the longitudinal tensile strength, longitudinal compressive strength, transverse tensile strength, transverse compressive strength, longitudinal shear strength, and transverse shear strength, respectively. ${\hat{\sigma }}_{11}$, ${\hat{\sigma }}_{22}$, and ${\hat{\tau }}_{12}$ refer to the in-plane normal and shear stresses (the 1-direction is aligned with fibers direction). The coefficient *α* defines the contribution of the shear stress to the fiber tensile failure initiation.

Equations (3)–(5) indicate that the conventional Hashin criterion does not take the delamination into account (there are modified versions of this criterion developed in the literature [26]; however, they require user-defined coding to implement in most FE packages). This is despite the fact that damage detection tests have confirmed the presence of delamination in several composite structures under impact. Figure 3 shows an example of the image obtained via X-ray microtomography technique (XMT) from the interior part of the impacted specimen in the present work. This image shows a cross-section 10 mm far from the impact center. The XMT reveals the presence of several delamination zones. Knowing that the conventional Hashin criterion cannot address the effect of delamination during numerical simulation, the difference between experimental and numerical results in Figure 2 can be partially explained.

The next question would be how significantly delamination can affect the global (effective) strengths of the composite. To this end, Soldatos and Shu [29] modeled perfectly and weakly bonded composite laminates. Their numerical results showed that the maximum capacity of weakly bonded laminates to carry tensile loads was declined. Colombo and Vergani [30] investigated the effect of delamination on the fatigue life of glass/epoxy composites and reported a 40% loss. Reis et al. [31] inserted Teflon layers at the middle of the plate thickness to make artificial delamination with different sizes (2 to 20 mm) in carbon/epoxy laminates and revealed that the delamination can reduce the tensile strength up to 17%, but surprisingly it did not affect Young's modulus. It was also shown in [25] that both strength and Young's modulus were independent of the size of delamination. The effect of delamination on the compressive behavior of glass fiber reinforced composites was investigated by Short et al. [32]. Artificial delaminated zones were made with PTFE films with different sizes (10 to 25 mm squares). Their work showed that the compression failure load decreases with increasing the size of delamination and also shifting the delamination zone towards the center of the laminate. A maximum strength reduction of 31% was reported for 25 mm^2^ delamination area placed at the center of the plate.

### 3.4. Effect of Fixture Geometry and Clamping Condition

The current literature also shows that the impact response of composites can be highly dependent on the clamping condition. Daiyan et al. [33] investigated the effect of clamping on impact response of samples made of 20% mineral (talc) and 80% elastomer modified polypropylene compound (ISO code PP + EPDM-TD20), a material that is being used in automotive exterior parts. The samples were put on a stand with a circular hole (40 mm diameter) at the center. Samples were tested with and without a circular (40 mm inner diameter) clamp. The reaction force that unclamped samples exerted to the striker was up to 10% higher than that of the clamped samples. This behavior could be attributed to the higher internal damage in the clamped samples. Very recently, Nilakantan and Nutt [10] also reported a similar trend of composite behavior. They performed both experimental and numerical analyses on the effect of clamping geometry on the impact response of soft body armors made of plain weave Kevlar fabrics. The advantages and disadvantages of six tested designs (Figure 5) were discussed in [10].

The comparison between the projectile deceleration after hitting the samples clamped in different configurations in Figure 5 showed that the maximum and minimum deceleration (proportional to the reaction force) were for the four-side held and diamond clamps, respectively [10]. The velocity of projectile corresponding to the circular clamp stood between the velocities of the other two clamping configurations. This would suggest that the more severe/bigger area the sample is constrained by the clamping system, the sooner the failure occurrence would be likely and the lower the peak force would be exerted to the projectile. If one compares the clamping condition of our experimental set-up (Figure 1(a)) to that of numerical simulation (Figure 1(b)), it is notable that the FE model assumes a fully clamped condition around the four sides of the sample, whereas in reality the tested samples would have had some degrees of freedom inside the fixture. This is mostly noticed when a sample slides out of the fixture under severe conditions (see Figure 6 for an example of such case when a soft-core sandwich panel was tested by the same test set-up and striker). In general, we could conclude that the numerical model, in contrast to reality, is modeled based on a fully clamped condition (with no degree of freedom in tied nodes) and as a result the predicted peak reaction force should be expected to be lower than experimental results. This brings the idea of applying a fourth correction factor (*η*_fix_ > 1) to mechanical properties to account for this clamping effect—a notion that has been rarely taken into account during the impact modeling of composites.

## 4. Proposed Simplified Modeling

With the background presented above in Sections 3.1–3.3 regarding the effects of four main factors on accuracy of simulation of composites, the task is now to incorporate these effects into an easy-to-implement macro-level FE model to predict impact response of the tested TWINTEX composite, specially assuming this is done by a user with no advanced skills on writing material subroutines.

### 4.1. Approach 1

In order to make a numerical model be able to capture experimental observations, the model input parameters should be carefully selected and, if needed, modified to reflect specific testing conditions and material behavior. This can be achieved by applying correction factors to the nominal (quasistatic) mechanical properties to bring in, for example, the effects of high strain rate, flexural bending, delamination, and clamping system. The following equation can be proposed to apply these effects on input materials properties, that is, elastic moduli, ultimate strengths, and fracture toughness values:

(6) $$\begin{array}{c} {\left(y_{\mathrm{mod}}\right)}_{i}={\left(y\times \eta_{\dot{\varepsilon }}\times \eta_{\text{ben}}\times \eta_{\text{del}}\times \eta_{\text{fix}}\right)}_{i}={\left(y\times \eta_{\text{total}}\right)}_{i}, \end{array}$$

where *y* and *y*_mod⁡_ are the original and modified material properties and $\eta_{\dot{\varepsilon }}$, *η*_ben_, *η*_del_, and *η*_fix_ are the correction factors for the strain rate, bending, delamination, and fixture clamping condition effects, respectively. These correction factors can be extracted from the literature if available. For the material under study, TWINTEX, the correction factors are extracted in Table 2 based on the available literature as follows.

**Figure 4:**
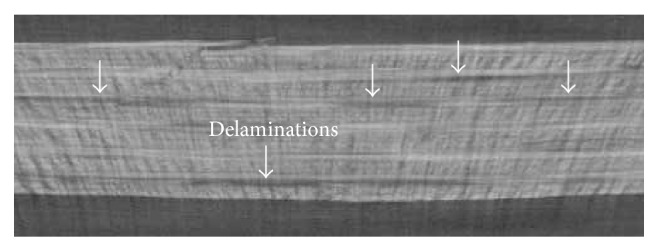
X-ray microtomography image of the impacted Twintex laminate (sample delamination zones are shown with arrows).

**Figure 5:**
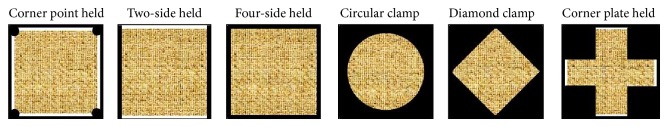
Six different clamping configurations compared during impact testing [10].

**Figure 6:**
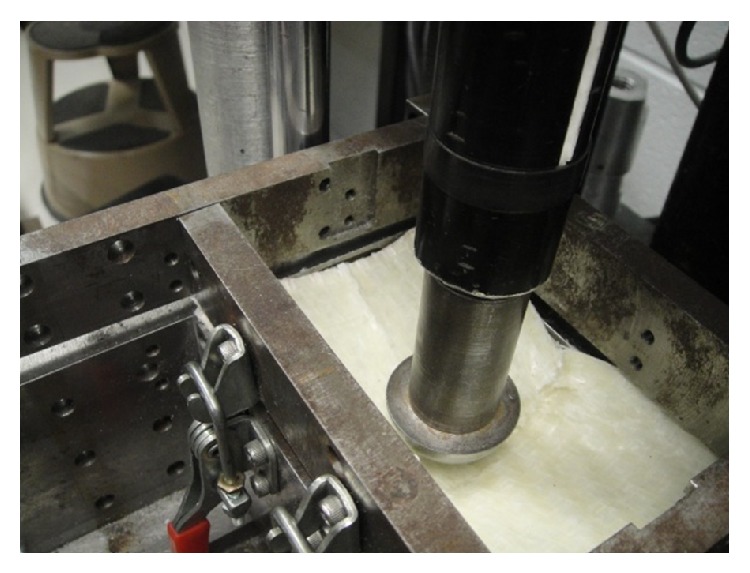
A soft-core sandwich panel slipped out of the fixture under 200 J impact (also notice the general high stress concentration pattern in Figure 1(b) near the fixture edge where the material slippage is seen in Figure 6).

According to the study by Santiuste [26] and their observation of 40% increase in composite strengths from tensile to bending mode, the correction factor for bending effect during impact simulation was set to 1.4. Short et al. [32] reported 17% and 31% deduction in tensile and compressive strengths, respectively, for completely delaminated laminates. Since such a severe large delamination was not observed in the X-ray microtomography of our samples (Figure 4), the corresponding correction factors were set at half of the effects reported in [27] as follows: 0.92 (i.e., 8% reduction) for the tensile strength, 0.85 (i.e., 15% reduction) for the compressive strength, and 0.70 for shear (i.e., 30% reduction, considering the shear as a more sensitive mode to delamination). Numerical simulation showed a wide range of strain rate on different elements during impact (0 to 1600 s^−1^). The average strain rate during whole impact event for all elements was found to be 63 s^−1^. Accordingly, this average strain rate was used to find the correction factor for strain rate effect from regression models in Figure 3 based on the work of Brown et al. [9]. For instance, the regression model in Figure 3 suggests compression strength of 313.33 MPa at the strain rate of 63 s^−1^. Hence, the compression strength correction factor was found by dividing the high strain rate property (313.33 MPa) by the quasistatic one (178.14 MPa), yielding 1.76. Also, a correction factor of 1.25 was set for the clamping condition effect (*η*_fix_ = 1.25) due to potentially slight freedom of samples in the fixture. Here, according to Table 2, an example of final modified property is given for the tensile strength: $X_{\text{corrected}}^{T}=X_{\text{quasi}-\text{static}}^{T}\times \eta_{\dot{\varepsilon }}\times \eta_{\text{ben}}\times \eta_{\text{del}}\times \eta_{\text{fix}}=288\times 2.124=611.7$ MPa. Results of the modified material properties are summarized in Table 3.

**Figure 7:**
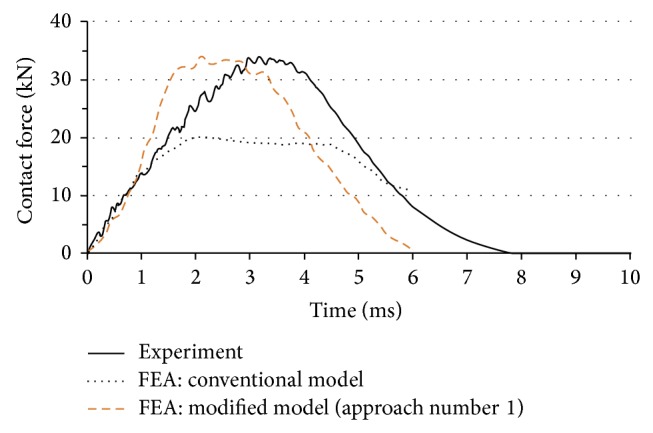
Comparison between experimental and two different numerical models; FEA with (i) quasistatic and (ii) modified material properties under Approach 1.

**Figure 8:**
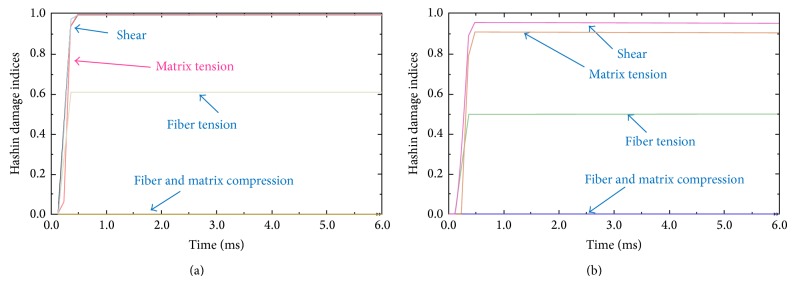
Progression of the failure on the impacted (top) face of the sample at (a) 1 mm and (b) 10 mm from the impact center.

Figure 7 compares the results of the modified model with the original one in Section 3 as well as the experimental data. This figure reveals considerable improvement in the prediction after applying the correction factors in Table 2.  Figure 8 illustrates the trend of damage on the impacted surface of the laminate via progression of the Hashin failure indices for two elements; (a) 1 mm and (b) 10 mm far from the impact center. The observed trends clearly show that failure due to the in-plane shear and matrix tensile cracking occurs close to the impact center (Figure 8(a)), shortly after the beginning of the event. The latter also supports the notion of applying the correction factors and changing the material properties for elements under impact from the early stages of the simulation, especially under high energy impact for the tested material and configuration.  Figure 8(b) shows that the element 10-mm far from the impact center is not completely failed (all index values are smaller than one). Figures 8(a) and 8(b) also indicate that the fiber and matrix compressive failures are predicted accurately (equal to zero) as the impacted face is under tension.

### 4.2. Approach 2

In general, the weak point about Approach 1 is that the user must estimate the average strain rate that the structure would experience under a given impact energy. Also the material properties at that specific strain rate should be accessible (via testing or published work on rate dependency of that particular material). Similarly, earlier estimations on bending mode, delamination, and clamping condition effects should be employed. However, if a material is new and only standard quasistatic data is available, a much simpler approach may be explored as follows.

**Figure 9:**
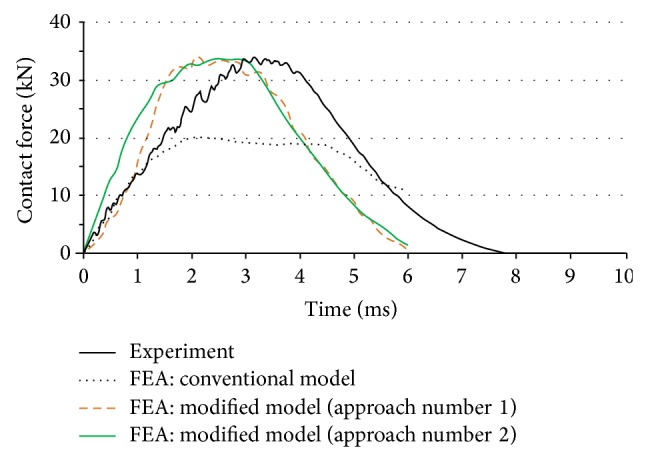
Comparison between experimental and three different numerical methods; (i) FEA with quasistatic properties, (ii) FEA with modified material properties under Approach 1, and (iii) FEA with modified material properties under Approach 2.

In Approach 2, one single correction factor is used for the entire set of quasistatic material properties (as opposed to Table 2 where different corrections factors were used for each property). This single correction factor for the twill weave Twintex laminate was estimated to be equal to 1.7.  Figure 9 shows the FEA result of this approach versus the earlier approaches and the experimental data. Surprisingly, the result of Approach 2 despite its simplicity by using one single correction factor is close to Approach 1, and both approaches show a reasonable predictability of maximum impact force. Although this agreement is favorable, some discrepancies can still be observed between the two simplified models and the experiment, especially if an analyst is interested in predicting the absorbed energy rather than the peak force. These discrepancies may be caused by the model assumptions made on (a) the sample or test conditions and (b) the material structural behavior. The former source refers to the difference between the assumed ideal sample configuration and the actual material condition (such as fiber misalignment, void content, inhomogeneity of material properties, etc.). Testing conditions refer to, for example, friction, the angle between impactor and sample, operator errors in fixture adjustment, and so on. Regarding the structural assumptions in the model, three main sources of discrepancy may be highlighted: (1) here the actual sample with a thickness of 6-mm has been modeled with shell elements; that is, with no normal stress along the impact direction, (2) fibers and the matrix are assumed to be perfectly bonded, (3) correction factors on mechanical properties are applied on all elements in the beginning of the simulation, whereas in the actual test condition, the time and location of each element (distance to the impact center) can affect magnitude of the corresponding correction factor; and finally (4) the micro/meso-level interaction of interlaced yarns was not taken into account.

## 5. Summary and Conclusion

In spite of significant advances in numerical simulation of complex structures under different types of loadings, a fully representative model is not yet developed to predict the impact response of fiber reinforced plastic composites (FRPs) under different test conditions. The reason for this challenge may be found in different intrinsic and extrinsic characteristics of composites, from anisotropic properties to uncontrolled parameters during sample fabrication and/or testing. Different FRP composites show different and sometime unexpected mechanical responses, especially under dynamic loads, that make their numerical simulations a bigger challenge. For instance, contrary to many other conventional materials, the experimental study [9] shows that the shear modulus and strength of glass reinforced polypropylene composite decrease at higher strain rates, or according to [31], delamination insert films did not affect Young's modulus of carbon/epoxy laminates, or the study [26] shows that the tensile material properties under bending deformation can be notably different from those under normal tensile loading (up to 40% difference). Similarly, results in recent works [10, 33] imply that the different clamping conditions during drop weight testing can be a large source of variation in the material response. Considering all these four factors, detailed numerical modeling of an FRP can be an obstacle for practitioners, either due to the lack of experimental data or due to the cost of advanced subroutines in conjunction to commercial FE packages. In this paper, as a preliminary tool, two simplified modeling approaches were discussed to make it possible to predict the impact force of a FRP composite using built-in models in commercial FE packages (here Abaqus).

To show the application of the two approaches, a glass fiber reinforced polypropylene composite was subjected to impact at 200 J and was simulated in Abaqus/Explicit. The built-in Hashin damage criterion was used for progressive damage of the material. The initial model using the material properties from quasistatic test data was far from the impact experimental data. To explain this deviation, X-ray microtomography was conducted on the samples and revealed delamination zones due to impact, which is not considered in the conventional Hashin failure criterion. The other source of this deviation was due to the difference between effective material properties in the tensile testing and the flexural testing (note that under out-of-plane impact, high speed bending is a dominant mode). Also, the quasistatic material properties are needed to be corrected for the strain rate dependency effect during impact. Finally, further to earlier works [10, 33], it was deemed that the difference between the actual clamping system during the drop weight test and its modeled boundary condition in simulations can be another source of deviation, which was addressed by applying a fourth correction factor to the mechanical properties. Results of the modified model showed that by correcting the quasistatic material parameters using four factors, the predictability of the numerical model is notably enhanced (Approach 1). In a second approach (Approach 2), only one overall correction factor was applied to the entire set of material properties. The first approach was relatively more accurate compared to test data; however, an estimation of average strain rate and the availability of literature on strain rate dependency mechanical properties, clamping effect, delamination sensitivity, and bending mode effect for a given material were needed. In contrast, the second method was found very straightforward to implement, and yet reasonably accurate, via iterating only a single constant correction factor.

It is believed that through similar future studies such correction factors on dynamic response of composites can be systematically explored and tabulated for different materials and test conditions and eventually can be used for simplified and fast simulations. Of course this effort should be taken as an interim solution as more advanced composite damage models are being developed and implemented in commercial FE packages.

## Figures and Tables

**Table 1:** Quasistatic mechanical properties of twill weave Twintex composites [12] (indices 1 and 2 refer to in-plane warp and weft directions and index 3 denotes the out-of-plane direction).

Properties (*y*_*i*_)	Values	
Tensile strength		
*σ*_11_	288	MPa
*σ*_22_	266	MPa
Compression strength		
*σ*_11_	155	MPa
*σ*_22_	150	MPa
Shear strength		
*τ*_12_	19	MPa
*τ*_21_	18	MPa
Modulus of elasticity		
*E*_11_	14	GPa
*E*_22_	13	GPa
Shear modulus		
*G*_12_	1.7	GPa
*G*_13_	1.8	GPa
*G*_23_	1.7	GPa
Poisson's ratio		
*υ*_12_	0.10	—
Fracture toughness		
*G*	220	kJ/m^2^

**Table 2:** Correction factors applied to the mechanical properties of the TWINTEX sample.

*y*_*i*_	Correction factors				
	Strain rate	Bending	Delamination	Fixture	Total
	$\eta_{\dot{\varepsilon }}$	*η*_ben_	*η*_del_	*η*_fix_	*η*_total_
Tensile strength and fracture toughness	1.32	1.40	0.92	1.25	2.124
Compression strength and fracture toughness	1.76	1.40	0.85	1.25	2.616
Shear strength and fracture toughness	0.71	1.40	0.70	1.25	0.866
Tensile modulus	1.04	1.00	1.00	1.25	1.306
Compression modulus	2.15	1.00	1.00	1.25	2.688
Shear modulus	0.19	1.00	1.00	1.25	0.233

**Table 3:** The ensuing modified material properties from Tables 1 and 2.

Modified properties (*y*_mod⁡,*i*_ = *y*_*i*_ × *η*_total,*i*_)	Values	
Tensile strength		
*σ*_11_	611.7	MPa
*σ*_22_	564.9	MPa
Compression strength		
*σ*_11_	405.5	MPa
*σ*_22_	392.5	MPa
Shear strength		
*τ*_12_	16.5	MPa
*τ*_21_	15.6	MPa
Modulus of elasticity		
*E*_11_	18.3	GPa
*E*_22_	17.0	GPa
Shear modulus		
*G*_12_	0.4	GPa
*G*_13_	0.4	GPa
*G*_23_	0.4	GPa
Poisson's ratio		
*υ*_12_	0.10	–-
Tensile fracture toughness		
*G*	467.2	kJ/m^2^
Compressive fracture toughness		
*G*	575.6	kJ/m^2^
